# Theoretical Analysis and Numerical Simulation of Ejection Demolding Process Parameters for Large Cylindrical Helical Gears After Die Forging

**DOI:** 10.3390/ma19102125

**Published:** 2026-05-19

**Authors:** Aihua Zhang, Guosheng Fei, Xiaoci Chen, Zuofa Liu, Jie Zhou, Yancheng Zhang, Jiansheng Zhang

**Affiliations:** 1 Sichuan Provincial Engineering Research Center of Aeronautical Material Testing and Die Forging Technology, College of Materials Engineering, Sichuan Polytechnic University, Deyang 618000, China; 2 Chongqing Key Laboratory of Advanced Mold Intelligent Manufacturing, College of Materials Science and Engineering, Chongqing University, Chongqing 400044, China; 3 Chongqing Jiepin Technology Co., Ltd., Chongqing 401329, China

**Keywords:** large cylindrical helical gear, gear die forging, ejection, numerical simulation, process parameters

## Abstract

To address the critical technical issue of difficult demolding following the die forging process of large cylindrical helical gears, a systematic theoretical analysis and process parameter investigation of the demolding technique for such forgings was conducted in the present work. Firstly, a mechanical theoretical model was established for the forging ejection and demolding procedure, and the influence mechanisms of friction coefficient and ejection velocity on ejection load, effective strain, and damage characteristics of the forging were quantitatively revealed. The results indicated that an increase in friction coefficient led to a remarkable growth in frictional resistance between the forging and the tooth-profile die cavity, which consequently elevated the maximum ejection load, effective strain, and damage value of the forging synchronously. Similarly, the maximum ejection load, peak effective strain, and maximum damage value of the forging increased sharply with the rise in ejection velocity. Therefore, it was proposed that in practical industrial production, the friction coefficient should be controlled within the range of 0.25 to 0.30 by adopting suitable high-temperature lubrication measures, and a relatively low ejection velocity should be preferentially adopted to guarantee the overall quality of the forged gear. This study provided a reliable theoretical basis and technical support for engineering applications. The optimized parameters (friction coefficient 0.25–0.30 and low ejection velocity) could be directly adopted in industrial production to reduce ejection load, lower strain and damage, and stabilize the forging quality of large cylindrical helical gears in actual die forging and demolding processes.

## 1. Introduction

In the die forging process of cylindrical helical gears, particularly for the high-strength steel or alloy metal materials, after undergoing high-temperature and high-pressure die forging deformation, the unique helical structure of the gears and the tight adhesion between the forgings and the tooth cavities of the dies rendered demolding difficult. This has emerged as a relatively prevalent technical challenge [1,2]. Such tight adhesion stemmed from the large forming forces during the die forging of cylindrical helical gears, which caused the tooth surfaces of the forgings to adhere closely to the die cavities and generated significant frictional resistance, thereby increasing the difficulty of separating the forgings from the tooth cavity dies [3,4].

To address this challenge, mold designers typically incorporate ejection mechanisms into die structures to facilitate the smooth demolding of helical gear forgings [5,6]. Conventional ejection systems, such as ejector pins, are actuated by the ejection cylinder of the forging press, which drives the ejector pins upward to separate the forging from the die cavity. Nevertheless, for the large helical gear forgings, with diameters exceeding 500 mm, the significant enlargement of geometric dimensions gives rise to a remarkable increase in the contact area with the tooth cavity die. This leads to stronger interfacial adhesion between the forging and the die cavity after forging, together with a more complicated distribution of contact stress, thereby easily inducing local adhesion and jamming, difficult demolding, as well as forming defects including tooth deformation of the forging and tooth damage of the die [7,8].

To overcome this challenge, researchers have developed various demolding methods to realize smooth demolding following the conventional die forging of cylindrical helical gears. Yoshida et al. [9] proposed a combined forming–demolding method for helical gear manufacturing. Under the action of the punch, the billet was extruded downward and gradually filled the tooth profile under the constraint of the die cavity. After the front section of the billet was formed, the punch continued to press downward, driving the subsequent workpiece to push the formed gear out of the die cavity. Liu [10] developed a through-type cold extrusion process for the fabrication of conventional internal helical gears. During the forming process, the upper punch pressed downward to force the billet to pass sequentially through the toothed lower punch cavity, thereby achieving full tooth profile filling. Through a series of optimized operations, this process ensured both forming quality and material utilization while reducing manufacturing costs. Chen [11] proposed a helical demolding method for conventional helical gear forgings, which employed an ejector pin with a helical groove milled along the helix direction of the gear. During demolding, the ejector pin rotated along the helical groove under the guidance of a pilot pin, thereby smoothly ejecting the die-forged helical gear. This design eliminated the need for complex demolding mechanisms and effectively resolved the demolding challenge of helical gears after die forging. Li [12] adopted thrust ball bearings to address the rotation issue during the demolding of conventional helical gear forgings. Owing to the helical angle structure of helical gears, relative rotation occurred between the tooth cavity die and the lower punch during demolding; installing a thrust ball bearing at the bottom of the lower punch reduced rotational resistance, enabling the lower punch to rotate synchronously and thus ensuring smooth demolding of the forging. Wu [13] integrated the die tooth cavity into the inner ring of a radial ball bearing, utilizing the rotational characteristics of the bearing during demolding to allow the forging to automatically rotate along the helical tooth cavity during ejection, thereby achieving smooth demolding of the helical gear. The aforementioned combined extrusion forming–through-type demolding process and helical demolding method have been widely applied to conventional cylindrical helical gear products and have yielded favorable demolding effects.

Although numerous studies have been conducted on the demolding of helical gears, most existing literature focuses on forming process optimization or demolding device improvement, with few studies systematically exploring the applicability and accuracy of theoretical analysis and numerical simulation in the demolding process of large cylindrical helical gear forgings after the hot die forging [14,15]. During the demolding process, friction conditions, demolding speed, and contact behavior between components directly affect the forging quality [16,17]. It has been verified in practice that the combination of theoretical models and numerical simulation can effectively predict the demolding force, stress distribution, and damage risk under different process parameters. Therefore, it was established a mechanical model to describe the demolding process in this study, analyzes the effects of process parameters (e.g., friction coefficient and demolding speed) on the demolding effect of helical gear forgings, and further verifies the reliability of theoretical analysis in evaluating the demolding behavior of large cylindrical helical gear forgings. The research findings can provide theoretical support for the process parameter design and quality control of large gear forgings.

## 2. Materials and Methods

### 2.1. Materials

The geometric parameters of the large cylindrical helical gear studied in this work are presented in Table 1. The gear part is fabricated from 18CrNiMo7-6 alloy steel, which possesses excellent properties such as high wear resistance and superior fatigue performance [18,19]. This alloy steel is widely utilized in critical components, including wind turbine gearbox gears, core aerospace structural parts, and heavy machinery transmission shafts [20]. The detailed chemical composition of 18CrNiMo7-6 alloy steel is summarized in Table 2.

### 2.2. Establishment of Finite Element Model

**Figure 1:**
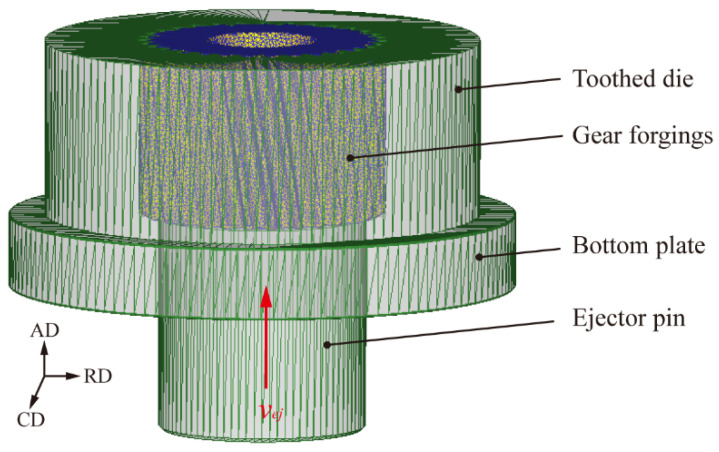
Finite element model of ejection process.

The forging and the tooth cavity die after the die forging were integrally inherent into the demolding process. The finite element model established for the demolding process is shown in Figure 1. The key parameters for numerical simulation of ejection demolding are listed in Table 3. The upward ejection speed of the ejector pin was defined as constant, while the tooth cavity die and the lower bolster plate remain fixed.

## 3. Results and Discussion

### 3.1. Theoretical Force Model of the Ejection Demolding Process

**Figure 2:**
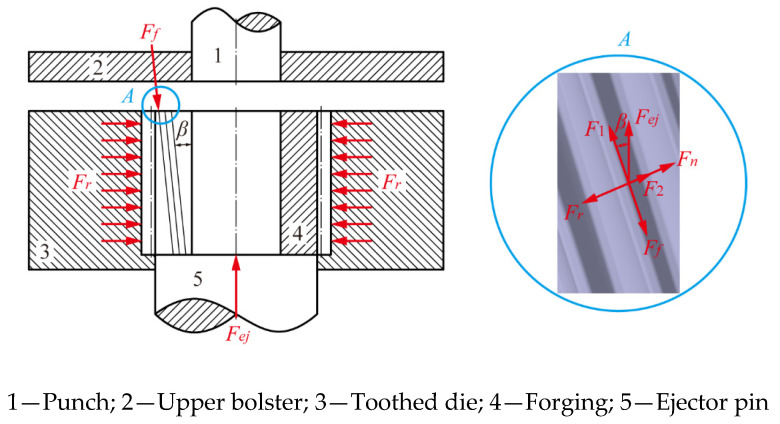
Schematic diagram of forces in ejection process.

After the forming process was completed, the forging was enclosed within a tool assembly consisting of a punch, an ejector pin, and a tooth cavity die. The ejector pin remained stationary during the forming stage and functions as an ejector after the forming. Figure 2 illustrates the force conditions acting on the large helical gear forging during the ejection demolding process, where *F_ej_* represents the vertically upward ejection force applied by the ejector pin. This ejection force could be decomposed into two components: *F*_1_ acting along the tooth direction, and *F*_2_ acting perpendicular to the tooth direction. Additionally, *F_r_* denotes the pressure exerted by the tooth cavity die on the forging, which is oriented perpendicular to the tooth surface. *F_f_* is the frictional force experienced by the gear teeth during demolding, directed downward along the helical tooth. *F_n_* represents the reaction force exerted by the forging teeth on the tooth cavity die, which is balanced within the forging as a whole.

Assuming that the forging is ejected at a constant velocity during demolding, the magnitude of the ejection force *F_ej_* can be derived using the following formulas:

(1) $$F_{1}=F_{ej}\cdot \cos\beta$$

(2) $$F_{2}=F_{ej}\cdot \sin\beta$$

(3) $$F_{f}=F_{1}$$

(4) $$F_{r}=F_{2}+F_{n}$$

(5) $$F_{f}=\mu F_{r}$$

Based on Equations (1)–(5), it follows that:

(6) $$F_{ej}=\frac{\mu F_{n}}{\cos\beta -\mu \sin\beta }$$

where *β* is the helix angle of the forging, which was set as 7.5° in this study; *μ* is the friction coefficient of the contact surface between the outer contour of the forging and the toothed die; and *F_n_* is the reaction force of the forging tooth profile against the cavity of the toothed die.

### 3.2. Effect of Key Ejection Process Parameters on the Demolding Quality of Forgings

During the ejection stage of large helical gear forgings, insufficiently precise control of process parameters may lead to irreversible deformation and damage to the formed forging, rendering it unable to meet technical requirements [21,22]. It can be seen from (6) that the ejection force *F_ej_* during demolding is influenced by the friction conditions and the reaction force *F_n_* of the forging tooth profile against the cavity of the toothed die. Furthermore, the reaction force *F_n_* is influenced by the ejection velocity.

#### 3.2.1. Influence of Friction Coefficient on Forging Demolding Quality

**Figure 3:**
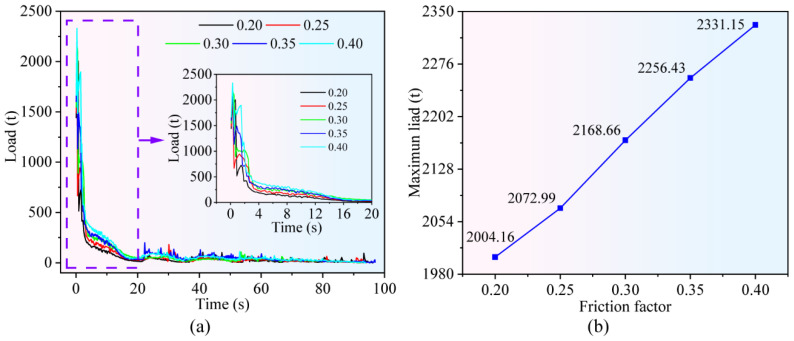
Influence of friction coefficient on ejection load: (**a**) time–load distribution curve; (**b**) curve of maximum ejection load variation with friction coefficient.

From Equation (6) can be derived that, for a given helix angle *β* of the forging and a fixed ejection process, the ejection force *F_ej_* is approximately proportional to the friction conditions. In this section, numerical simulations were performed with friction coefficients of 0.20, 0.25, 0.30, 0.35, and 0.40, while the upward ejection velocity of the ejector pin was set at 5 mm/s, and other process parameters remained unchanged. Figure 3a presents the ejection load variation curves corresponding to different friction coefficients. It could be observed that the ejection load reached its maximum at the initial stage of demolding, and gradually decreased to zero as the forging was progressively separated from the toothed die.

Figure 3b presents the variation curve of the maximum ejection load versus the friction coefficient during the demolding process. As the friction coefficient increases, the maximum ejection load shows a gradual upward trend, which is generally consistent with the trend predicted by Equation (6). When the friction coefficient is 0.40, the maximum ejection load during demolding increases to 2331.15 t, representing a 16.3% increase compared with that at a friction coefficient of 0.20. This phenomenon can be attributed to the fact that an increase in the friction coefficient leads to a corresponding increase in the frictional resistance between the forging and the tooth-profile die cavity, thereby resulting in a gradual rise in the required ejection load.

**Figure 4:**
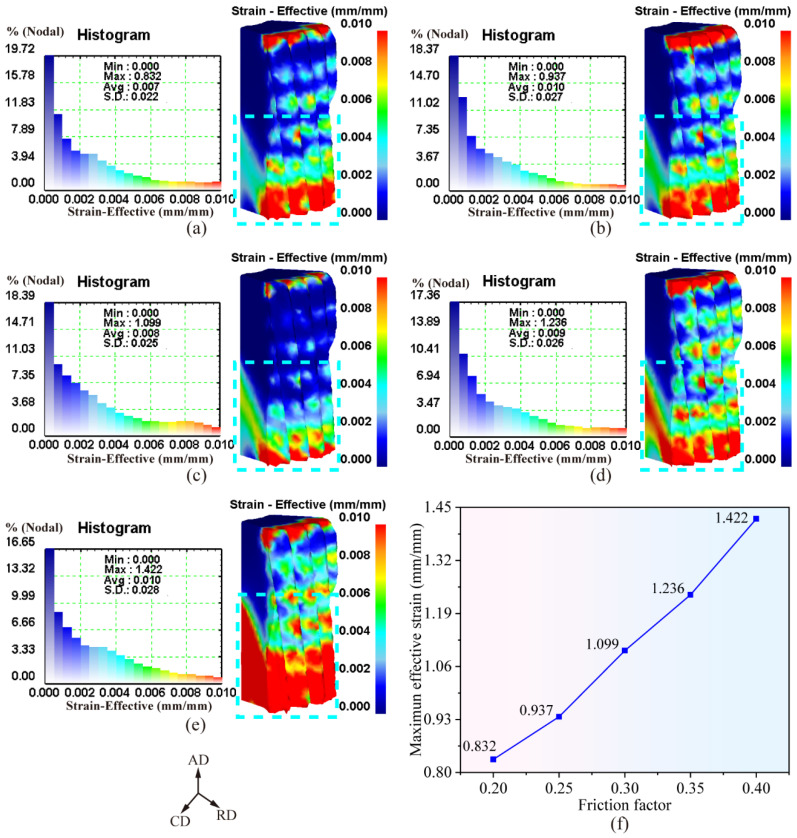
Effective strain distribution law of forgings with different friction coefficients: (**a**–**e**) contour maps of effective strain distribution for friction coefficients of 0.20~0.40; (**f**) variation curve of maximum effective strain with friction coefficient.

Figure 4a–e presents the effective strain distribution of the forging after demolding under different friction coefficients. It can be observed that the friction coefficient exerts a significant influence on the effective strain distribution of the forging post-demolding. With an increase in the friction coefficient, the small-strain region (blue area within the cyan short-dashed box) at the lower end face of the forging gradually shrinks, while the large-deformation region (red area within the cyan short-dashed box) expands progressively. This phenomenon is attributed to the enhanced frictional resistance between the forging and the die cavity as the friction coefficient increases, which intensifies the local stress concentration and thus leads to a broader range of high-strain areas in the forging.

Figure 4f depicts the variation in the maximum effective strain of the forging as a function of the friction coefficient. It can be seen that the maximum effective strain of the forging after demolding increases gradually with rising friction coefficient. This behavior can be attributed to the elevated frictional resistance imposed by the tooth-profile die cavity on the forging surface during demolding. The increased interfacial resistance forces the surface material of the forging to overcome greater constraints during ejection, resulting in a progressive increase in the effective strain.

The damage generated during the demolding process of the forging directly affects the tooth profile accuracy and surface quality, and is critical to determining the final performance of large helical gear forgings. To accurately quantify and analyze the damage evolution law during the ejection stage, this study introduces the Johnson–Cook damage model developed by Wu et al. [23] for 18CrNiMo7-6 alloy steel. This model is applied to the damage evaluation of large helical gear forgings after demolding, providing a reliable theoretical basis for the subsequent optimization of the ejection process and the reduction in tooth profile damage.

**Figure 5:**
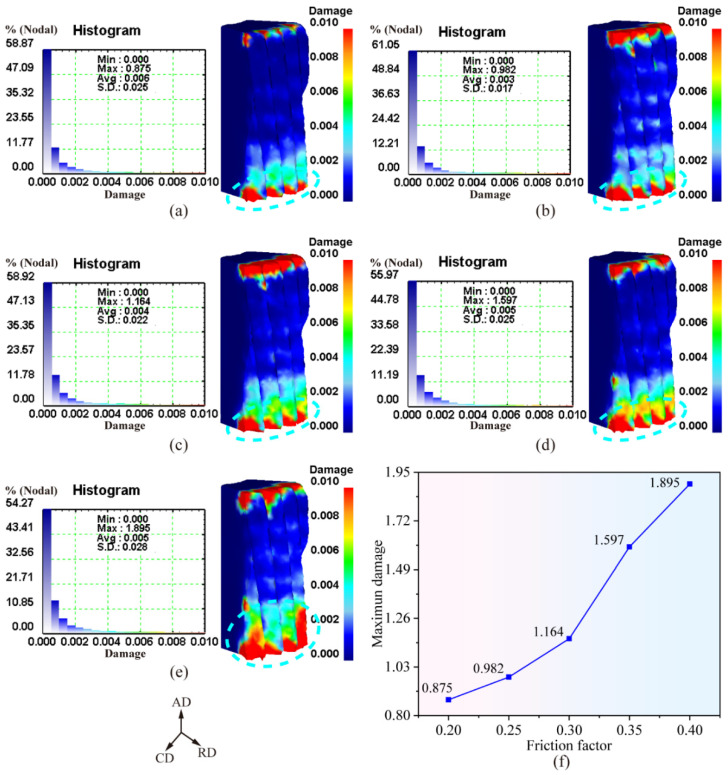
Damage distribution law of forgings with different friction coefficients: (**a**–**e**) contour maps of damage distribution for friction coefficients of 0.20~0.40; (**f**) variation curve of maximum damage with friction coefficient.

Figure 5a–e shows the damage distribution of the forging after demolding under various friction coefficients. It can be observed that the friction coefficient exerts a significant influence on the damage distribution within the forging. With an increase in the friction coefficient, the high-damage region (marked by the cyan short-dashed box) at the tooth profile on the lower end face of the forging gradually expands, indicating that the overall damage level of the forging increases progressively with rising friction coefficient.

Figure 5f plots the variation in the maximum damage value of the forging as a function of the friction coefficient. The maximum damage value after demolding is seen to increase monotonically with increasing friction coefficient. This behavior arises from the enhanced frictional resistance imposed by the tooth-profile die cavity on the surface layer of the forging, which intensifies the material damage during the demolding process.

In summary, an increase in the friction coefficient elevates the frictional resistance between the forging and the tooth-profile die cavity, thereby giving rise to a higher ejection load. Meanwhile, the intensified interfacial friction exerted on the surface layer of the forging leads to a gradual rise in both the effective strain and damage level of the material during the demolding process.

#### 3.2.2. Influence of Ejection Velocity on Forging Demolding Quality

According to Equation (6), when the helix angle *β* of the forging is set to a specific value and the friction conditions during the demolding process remain constant, the ejection force *F_ej_* is approximately proportional to the reaction force *F_n_* exerted by the forging tooth profile on the cavity of the toothed die. Moreover, the ejection velocity has a significant influence on the reaction force *F_n_*. In this section, numerical simulations were conducted with upward ejection velocities of the ejector pin set at 1 mm/s, 5 mm/s, 10 mm/s, 15 mm/s, and 20 mm/s, while the friction coefficient was maintained at 0.30 and other process parameters remain unchanged.

**Figure 6:**
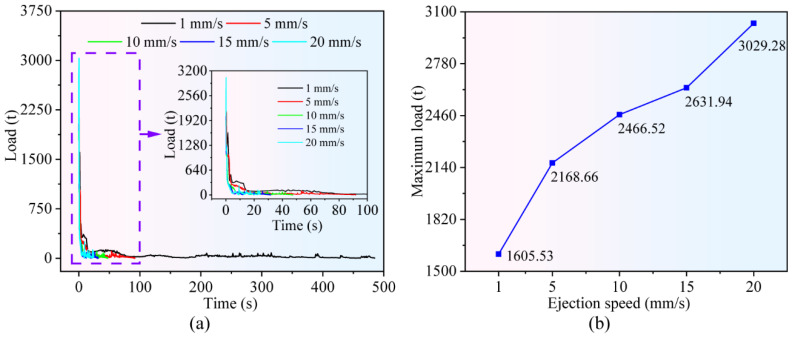
Effect of ejection speed on ejection load: (**a**) time–load distribution curve; (**b**) variation curve of maximum ejection load with ejection speed.

Figure 6a shows the variation curves of ejection load under different ejection velocities. It can be seen that the ejection load peaks at the initial stage of demolding. This is attributed to the largest contact area between the forging and the die at this moment, accompanied by the maximum frictional resistance that needs to be overcome. As the forging is gradually separated from the tooth-profile die, the contact area diminishes, and the frictional force decreases accordingly, resulting in a gradual decline in the ejection load until it finally approaches zero. Furthermore, the ejection velocity of the ejector pin exerts a considerable influence on the duration of the demolding process. A higher ejection velocity corresponds to a shorter demolding duration. At an ejection velocity of 20 mm/s, the complete demolding process of the forging takes only 24.25 s.

Figure 6b further illustrates the relationship between the ejection velocity and the maximum ejection load during the demolding process. As the ejection velocity increases, the maximum ejection load shows a significant upward trend. When the ejection velocity reaches 20 mm/s, the maximum ejection load increases to 3029.28 t, representing an 88.7% increase compared with that at an ejection velocity of 1 mm/s.

**Figure 7:**
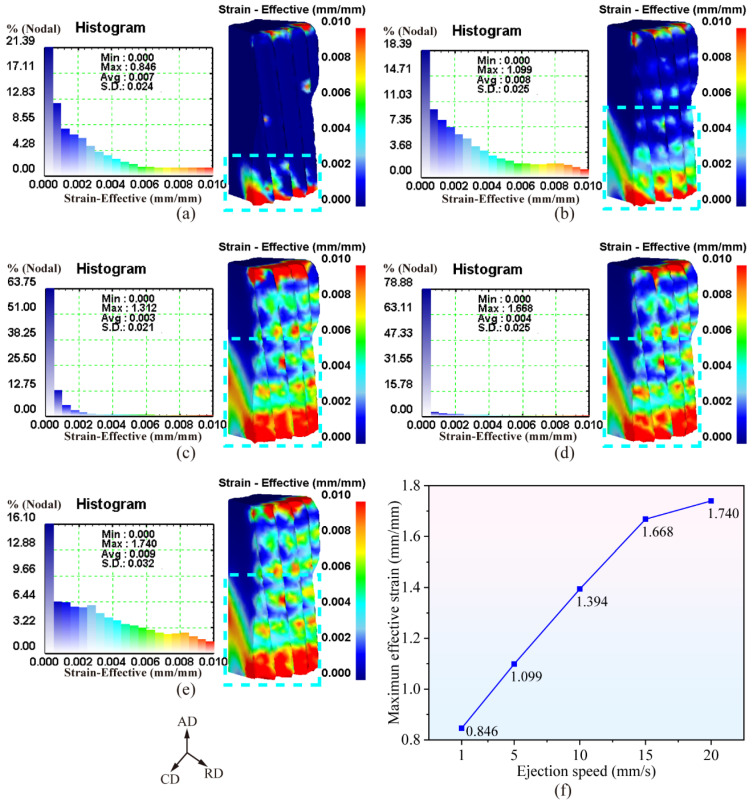
Effective strain distribution law of forgings with different ejection speeds: (**a**–**e**) contour maps of effective strain distribution for ejection speeds of 1~20 mm/s; (**f**) variation curve of maximum effective strain with ejection speeds.

Figure 7a–e illustrates the effective strain distribution of the forging after demolding under different ejection velocities. It can be observed that the ejection velocity exerts a significant influence on the pattern of effective strain distribution. As the ejection velocity increases, the small-strain region (blue area within the cyan dashed box) on the lower end face of the forging gradually shrinks, while the large-deformation region (red area within the cyan dashed box) expands progressively.

Figure 7f presents the variation curve of the maximum effective strain of the forging with respect to the ejection velocity. It is evident that the maximum effective strain of the forging after demolding increases gradually as the ejection velocity rises. This phenomenon can be attributed to the fact that a higher ejection velocity leads to a more significant impact load exerted by the punch on the forging, which transfers more kinetic energy to the forging in a short period. Under such conditions, the dislocation motion rate within the metal increases; meanwhile, the extremely short deformation time limits heat dissipation, resulting in reduced flow stress and enhanced metal plasticity, thereby facilitating plastic deformation and increasing the effective strain.

**Figure 8:**
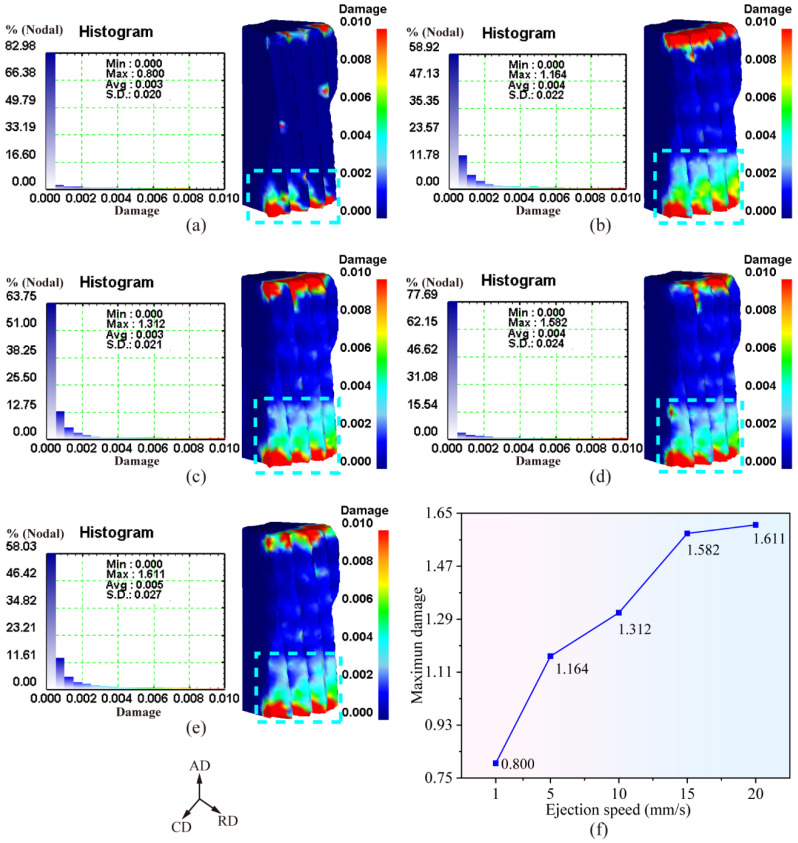
Damage distribution law of forgings with different ejection speeds: (**a**–**e**) contour maps of damage distribution for ejection speeds of 1~20 mm/s; (**f**) variation curve of maximum damage with ejection speed.

Figure 8a–e displays the damage distribution of the forging after demolding under different ejection velocities. It can be observed that ejection velocity exerts a significant influence on the damage distribution pattern within the forging. The high-damage region (marked by the cyan short-dashed box) at the tooth profile on the lower end face of the forging gradually expands, indicating that the overall damage level of the forging increases progressively with rising ejection velocity.

Figure 8f plots the variation in the maximum damage value of the forging as a function of ejection velocity. It is clearly seen that the maximum damage value after demolding increases monotonically with increasing ejection velocity. When the ejection velocity reaches 20 mm/s, the maximum damage value rises to 1.611, representing an increase of 101.4% compared with that at an ejection velocity of 1 mm/s.

In summary, with increasing ejection velocity, the maximum ejection load exhibits a gradually increasing trend, and both the effective strain and damage of the forging after demolding also increase progressively.

## 4. Conclusions

In this study, theoretical analysis and numerical investigation were performed to address the demolding challenges encountered in the hot die forging of large cylindrical helical gear forgings. The main conclusions are summarized as follows:

(1) A mechanical theoretical model for the ejection demolding process of large cylindrical helical gears was established. It is confirmed that ejection force is significantly affected by friction coefficient and ejection velocity.
(2) With the increase in friction coefficient from 0.20 to 0.40, the maximum ejection load increases by 16.3%, the maximum effective strain and damage value also rise synchronously. To ensure the forging quality and die service life, the friction coefficient can be stabilized within 0.25–0.30 by adopting high-temperature graphite lubricant, glass lubricant, or molybdenum disulfide-based composite lubricant with suitable coating thickness and uniform spraying process.
(3) As the ejection velocity increases from 1 mm/s to 20 mm/s, the maximum ejection load increases by 88.7%, and the maximum damage value increases by 101.4%, indicating that high ejection velocity significantly aggravates strain and damage. Therefore, a relatively low ejection velocity is preferred to reduce impact and ensure gear dimensional accuracy and surface quality.
(4) The theoretical and numerical results obtained in this study can be used to predict and optimize ejection demolding behavior, which demonstrates the applicability and accuracy of theoretical analysis in the damage evaluation of large cylindrical helical gears during the demolding process. These findings can be directly applied to practical production by adopting high-temperature graphite or glass-based lubricants with reasonable coating and spraying processes to control the friction coefficient within 0.25–0.30, setting a low ejection velocity on the press equipment to reduce impact and damage, and applying the established mechanical model to predict ejection force and optimize the die structure before production, so as to transform numerical analysis results into practical and reliable process guidelines for the die forging and demolding of large cylindrical helical gears.

## Figures and Tables

**Table 1:** Key parameters of the large cylindrical helical gear part.

	Value	Parameter	Value
Number of teeth	31	Tip diameter (mm)	634.40
Normal module (mm)	19.10	Root diameter (mm)	573.08
Normal pressure angle (°)	22.50	Pitch diameter (mm)	597.21
Helix direction	Left	Bore diameter (mm)	330.00
Helix angle (°)	7.50	Face width (mm)	450.00

**Table 2:** Chemical composition of 18CrNiMo7-6 steel (mass fraction, %).

C	Si	Mn	P	S	Cr	Mo	Ni	Fe
0.15~0.21	≤0.40	0.50~0.90	≤0.025	≤0.035	1.50~1.80	0.25~0.35	1.40~1.70	Bal.

**Table 3:** Key parameters for numerical simulation of ejecting a large cylindrical helical gear.

Parameter	Setting	Parameter	Setting
Billet material	18CrNiMo7-6	Ambient temperature	30 °C
Die material	AISI H13	Ejection speed	1~20 mm/s
Billet type	Plastic	Thermal radiation coefficient	0.7
Die type	Rigid	Thermal convection coefficient	0.02 N/s/mm/°C
Billet mesh number	200,000	Thermal conduction coefficient	5 N/s/mm/°C
Billet heating temp.	1250 °C	Friction coefficient	0.2~0.4
Die preheating temp.	200 °C	Friction type	Shear

## Data Availability

The original contributions presented in this study are included in the article. Further inquiries can be directed to the corresponding authors.
